# Refining Lung Cancer Diagnosis and Staging with Bronchoscopy and EBUS-TBNA: Evidence from a Regional Romanian Study

**DOI:** 10.3390/medicina61091528

**Published:** 2025-08-26

**Authors:** Mihai Olteanu, Natalia Motaș, Gabriela Marina Andrei, Virginia Maria Rădulescu, Nina Ionovici, Marius Bunescu, Daniela Luminița Zob, Veronica Manolache, Corina Budin, Florentina Dumitrescu, Viorel Biciușcă, Ramona Cioboată

**Affiliations:** 1 Department of Internal Medicine—Pneumology, Faculty of Medicine, University of Medicine and Pharmacy from Craiova, 200349 Craiova, Romania; mihai.olteanu@umfcv.ro (M.O.);; 2 Department of Thoracic Surgery II, Faculty of Medicine, “Carol Davila” University of Medicine and Pharmacy, 022328 Bucharest, Romaniaveronicamanolache@gmail.com (V.M.); 3 Department of Medical Informatics and Biostatistics, Faculty of Medicine, University of Medicine and Pharmacy of Craiova, 200349 Craiova, Romania; 4 Department of Occupational Medicine, Faculty of Medicine, University of Medicine and Pharmacy from Craiova, 200349 Craiova, Romania; 5 Department of Medical Oncology II, “Prof. Dr. Al. Trestioreanu” Institute of Oncology, 022328 Bucharest, Romania; 6 Department of Pathophysiology, George Emil Palade University of Medicine, Pharmacy, Science, and Technology of Târgu Mureș, 540139 Târgu Mureș, Romania; 7 Department of Infectious Diseases, Faculty of Medicine, University of Medicine and Pharmacy from Craiova, 200349 Craiova, Romania

**Keywords:** bronchoscopy, interventional techniques, lung cancer, environmental & occupational health, epidemiology

## Abstract

*Background*: Lung cancer remains the leading cause of cancer-related mortality worldwide. Timely and accurate diagnosis and staging are crucial for treatment decisions. *Objective*: To assess the feasibility, safety, and diagnostic/staging yield of a bronchoscopy-based pathway supported by EBUS-TBNA in a regional Romanian center. Bronchoscopy combined with endobronchial ultrasound-guided transbronchial needle aspiration (EBUS-TBNA) may reduce the need for surgical confirmation, yet its implementation in regional centers is inconsistent. *Materials and Methods*: This retrospective study included 67 patients with suspected lung cancer evaluated at a regional oncology center between December 2023 and February 2024. All patients underwent bronchoscopy, and EBUS-TBNA was performed in those with mediastinal lymphadenopathy on imaging, with endoscopic tissue biopsies (endobronchial/EBUS-TBNA). Demographic, clinical, histological, and molecular data were collected and analyzed using descriptive statistics and chi-square/Fisher’s exact tests. *Results*: Among the 67 patients, 42 (62.7%) underwent EBUS-TBNA. The majority were diagnosed in advanced stages (stage III–IV: 83.6%), with adenocarcinoma being the most frequent histological subtype. PD-L1 expression was positive in 52.2% of cases, and p63 in 67.2%. No significant procedural complications occurred, and adequate tissue sampling for histopathological and molecular analyses was achieved in all cases. Associations were found between PD-L1 and advanced TNM stage (*p* = 0.026), as well as between p63 status and TNM stage (*p* = 0.002). *Conclusions*: This study supports the feasibility and safety of a bronchoscopy-based diagnostic and staging algorithm supported by EBUS-TBNA, achieving reliable sampling and avoiding surgical confirmation in a regional oncology setting. Further prospective studies are warranted to validate these findings.

## 1. Introduction

Lung cancer remains the leading cause of cancer-related mortality worldwide, with over 2.2 million new cases and 1.8 million deaths each year, as described by Sung et al. [1]. Although incidence and mortality have declined in some high-income countries due to tobacco control and screening, the burden remains high or even rising in Eastern Europe, according to Schabath and Cote [2]. Multiple environmental and occupational exposures, such as radon and asbestos, further contribute to lung cancer risk (Islami et al. [3]).

In the 2021 WHO classification, Travis et al. [4] highlighted the importance of dividing lung cancer into non-small cell lung cancer (NSCLC; adenocarcinoma, squamous cell carcinoma, large cell carcinoma) and SCLC, along with neuroendocrine tumors. The therapeutic landscape has advanced with targeted therapies against epidermal growth factor receptor (EGFR), anaplastic lymphoma kinase (ALK), ROS1, and others, and with immunotherapy guided by PD-L1 expression, as discussed by Lindeman et al. [5] and Herbst et al. [6]. In NSCLC, the integration of immunotherapies, particularly immune checkpoint inhibitors targeting the PD-1/PD-L1 pathway, has led to significant and sustained responses in patients without targetable oncogenic driver alterations. EGFR plays a pivotal role in the pathogenesis of lung cancer, particularly in NSCLC. EGFR is a transmembrane tyrosine kinase receptor that regulates cell proliferation, survival, and differentiation through downstream signaling pathways such as phosphatidylinositol 3-kinase/protein kinase B (PI3K-AKT), mitogen-activated protein kinase (MAPK), and the Janus kinase/Signal transducer and activator of transcription (JAK-STAT) [7]. Accurate staging remains critical, traditionally based on the tumor, node, metastasis (TNM) system, with further updates in the ninth edition described by Detterbeck et al. [8].

Liquid biopsy—most commonly the analysis of circulating tumor DNA (ctDNA) from peripheral blood—offers a non-invasive, repeatable approach to detect actionable variants and to monitor clonal evolution. It can shorten time-to-treatment and preserve limited tissue for histology and immunohistochemistry; however, tissue sampling remains the diagnostic standard in suspected lung cancer. In this study, all diagnoses and biomarker assessments were performed on endoscopically obtained tissue.

Bronchoscopy has been a cornerstone of diagnosis for decades, enabling visualization and tissue sampling under local anesthesia, as summarized by Criner et al. [9]. In the last 15 years, endobronchial ultrasound-guided transbronchial needle aspiration (EBUS-TBNA) has revolutionized mediastinal staging, replacing mediastinoscopy in many countries, with strong evidence from Evison et al. [10]. Biondini et al. [11] reviewed recent technical innovations that further improve tissue yield and safety.

However, Steinhauser Motta et al. [12] reported that in many regional centers, including parts of Eastern Europe, bronchoscopy and EBUS-TBNA remain underutilized, and patients still undergo unnecessary surgical procedures for confirmation, delaying treatment initiation. Dunne et al. [13] emphasized the opportunity to adopt minimally invasive algorithms to optimize time, costs, and patient safety.

Therefore, this study aimed to evaluate the feasibility, safety, and preliminary performance of a bronchoscopy-based diagnostic and staging pathway, supported by EBUS-TBNA, in a regional Romanian oncology setting.

## 2. Materials and Methods

### 2.1. Study Design and Setting

This retrospective study was conducted at the Saint Nectarie Oncology and Radiotherapy Center in Craiova, Romania, between December 2023 and February 2024.

### 2.2. Analysis Cohort

Adults (≥18 years) evaluated for suspected lung cancer without histopathological confirmation at presentation who underwent bronchoscopy—with selective EBUS-TBNA when mediastinal nodal disease was suspected—and with complete clinical/procedural records were included. Patients with missing key variables were excluded from the analysis. The EBUS-TBNA subgroup comprised patients with suspected mediastinal nodal involvement (short-axis ≥ 10 mm on CT and, when available, PET-avid nodes) who underwent EBUS-TBNA for tissue confirmation and mediastinal staging.

### 2.3. Exclusion Criteria

Exclusion criteria comprised severe coagulation disorders, unstable cardiovascular or neurological disease, severe psychiatric disorders, refusal to consent to the proposed procedures, or missing key clinical/procedural data.

### 2.4. Procedures

All patients underwent an initial clinical evaluation and chest imaging (chest X-ray and/or thoracic computed tomography (CT)). Flexible bronchoscopy was performed in all cases. Procedures were carried out under local anesthesia with mild sedation according to the institutional protocol. Topical anesthesia included lidocaine administered by spray to the oropharynx and by aliquots via the working channel as required, and mild sedation was administered under anesthesiology supervision according to the institutional protocol. Supplemental oxygen was delivered via low-flow nasal cannula; high-flow nasal cannula (HFNC) or a laryngeal mask airway (LMA) were available for selected higher-risk patients at the discretion of the bronchoscopist and anesthesia team. Procedural suitability (ability to tolerate topical anesthesia and mild sedation) was assessed by institutional protocol. EBUS-TBNA was performed selectively in patients with mediastinal lymphadenopathy larger than 10 mm on CT scans as per the center’s practice. Pre-procedure PET-CT was not performed systematically; when available, it informed the selection of PET-avid nodal targets. When EBUS-TBNA was undertaken, nodal stations were sampled starting with the highest expected stage (N3/N2), and additional stations were sampled if safe and feasible. A minimum of three needle passes per targeted nodal station were attempted when safe. Biopsy specimens were collected for histopathology, immunohistochemical analysis (PD-L1, p63), and, when adequate, molecular testing (EGFR). In cases where the first sample was inconclusive, repeat bronchoscopy or EBUS was performed. All samples represented tissue biopsies obtained endoscopically: endobronchial forceps biopsies and/or EBUS-TBNA nodal cores/aspirates processed as tissue. Cytological fluid was not used for diagnostic purposes in this cohort.

### 2.5. Pathology and Molecular Methods

All tissue was fixed in 10% neutral buffered formalin, paraffin-embedded, and sectioned at 3–4 µm for hematoxylin–eosin (H&E) and immunohistochemistry (IHC). IHC was performed on formalin-fixed, paraffin-embedded (FFPE) sections with heat-induced epitope retrieval and a polymer-based detection system with diaminobenzidine (DAB); appropriate positive and negative controls were run with each batch. PD-L1 was reported as Tumor Proportion Score (TPS, % tumor cells with membranous staining). p63 was used to support squamous differentiation (nuclear staining), thyroid transcription factor-1 (TTF-1) to support pulmonary origin (nuclear staining), synaptophysin (SYN) to confirm neuroendocrine differentiation (granular cytoplasmic staining), and the Ki-67 proliferation index was expressed as the percentage of positive tumor nuclei.

For molecular testing, when tissue adequacy permitted, EGFR mutational analysis was performed on FFPE material according to local laboratory protocols (DNA extraction followed by targeted real-time PCR or next-generation sequencing). Results were summarized at the exon level (18–21) in the Results section.

### 2.6. Safety Endpoints

Safety was defined a priori as: (i) absence of clinically significant procedure-related events (clinically relevant bleeding, pneumothorax requiring tube drainage, hypoxemia requiring escalation, hemodynamic instability, or need for advanced airway/support); (ii) adequate tolerance of local anesthesia and mild sedation without escalation; and (iii) successful acquisition of tissue sufficient for histopathological and, when adequate, molecular analyses.

### 2.7. Data Collection

The collected data included demographic characteristics, risk factors (such as smoking history and occupational exposure), symptoms, final TNM stage, histopathological subtype, molecular markers, and relevant laboratory results.

### 2.8. Statistical Analysis

Primary data were first organized and categorized for statistical analysis using Microsoft Excel 2021 (Microsoft Corporation, Redmond, WA, USA). Statistical analysis was subsequently performed with IBM SPSS Statistics for Windows, version 26.0 (IBM Corp., Armonk, NY, USA). Continuous variables were summarized as mean ± standard deviation (SD) for normally distributed data or as median with interquartile range (IQR) for non-normally distributed data. The distribution of continuous variables was assessed using the Shapiro–Wilk test and visual inspection of histograms. Categorical variables were presented as absolute numbers and relative frequencies (%). Associations between categorical variables were tested using the Chi-square test or Fisher’s exact test, as appropriate. Comparisons of continuous variables across groups were performed using the Kruskal–Wallis test when the assumption of normality was not met. Statistical significance was defined as a two-tailed *p*-value < 0.05, with results interpreted within a 95% confidence interval.

### 2.9. Ethical Considerations

The study was approved by the institutional ethics committee (University of Medicine and Pharmacy of Craiova; approval no. 26/720/1/20.07.2023) and conducted in accordance with the Declaration of Helsinki. Written informed consent was obtained at the time of care for all clinical procedures; the retrospective analysis of de-identified data was approved by the ethics committee and did not require additional consent.

## 3. Results

### 3.1. Patient Characteristics

In the study group of 67 patients, the majority were male (n = 40, 59.7%), compared to 27 female patients (40.3%). Most patients originated from urban areas (61.2%, n = 41), versus rural areas (38.8%, n = 26). Active or former smokers accounted for 43 cases (64.2%), while 24 patients (35.8%) were non-smokers. Occupational exposure to potential carcinogens was reported in 40 patients (59.7%), with 27 (40.3%) having no such exposures. Full details of these distributions are summarized in Table 1.

Regarding comorbidities, 14 patients (20.9%) had no associated diseases, while COPD was the most frequent comorbidity (n = 23, 34.3%), followed by heart disease (n = 13, 19.4%), diabetes mellitus (n = 10, 14.9%), and bronchial asthma (n = 7, 10.4%).

### 3.2. Tumor Staging and Histological Subtypes

According to TNM staging, 42 patients (62.7%) were diagnosed in stage IV, 14 patients (20.9%) in stage III, 6 patients (9.0%) in stage II, and 5 patients (7.5%) in stage I, as seen in Table 2. The histopathological subtypes were distributed as follows: adenocarcinoma (subtype 1) in 25 patients (37.3%), squamous carcinoma (subtype 2) in 15 patients (22.4%), small cell carcinoma (subtype 3) in 14 patients (20.9%), and neuroendocrine tumors (subtype 4) in 13 patients (19.4%). By diagnostic group, G1a included 27 patients (40.3%), G1b included 15 patients (22.4%), G2 included 14 patients (20.9%), and G3 included 11 patients (16.4%).

### 3.3. Procedures and Feasibility

**Figure 1:**
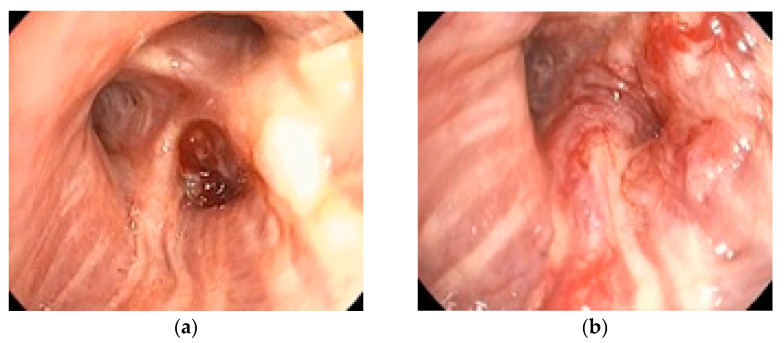
Bronchoscopic image (institutional archive): (**a**) 12 December 2023; (**b**) 16 January 2024.

**Figure 2:**
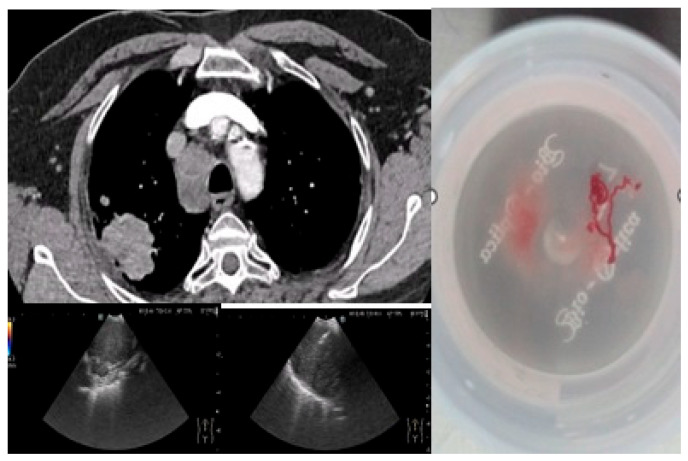
EBUS-TBNA image (institutional archive).

All patients (100%) underwent bronchoscopy, while EBUS-TBNA was performed in 42 patients (62.7%) with mediastinal lymphadenopathy detected on imaging. No significant complications related to either procedure were reported, confirming the safety of the proposed algorithm. Figure 1 illustrates a representative bronchoscopic finding in a 72-year-old ex-smoker male with an adenocarcinoma of the dorsal segment of the right upper lobe, showing well-vascularized tissue with distal bronchial invasion and significant stenosis due to extrinsic compression and mucosal infiltration. Figure 2 depicts an EBUS-TBNA procedure in a 69-year-old ex-smoker male without endobronchial tumor expression, performed under local anesthesia and mild sedation at station 4R, with a final diagnosis of small cell carcinoma confirmed by immunohistochemistry (thyroid transcription factor-1 (TTF1) positive, synaptophysin (SYN) positive, Ki67 proliferation index 98%).

Endoscopic sampling ultimately yielded a definitive histopathological diagnosis in all patients (67/67). Among those undergoing EBUS-TBNA (42/67, 62.7%), eight procedures (19.0%) were repeated to secure sufficient tissue for immunohistochemistry and/or molecular testing.

Procedurally, all patients underwent bronchoscopy (67/67, 100%), and EBUS-TBNA was performed in 42/67 (62.7%) for confirmation and mediastinal staging. Pre-procedure PET-CT was not performed systematically; when available, it guided the selection of PET-avid nodal targets. Rapid on-site cytologic evaluation (ROSE) was not routinely available. When EBUS-TBNA was undertaken, nodal stations were prioritized from the highest expected stage (N3/N2) downward when safe and feasible, and a minimum of three passes per targeted nodal station were attempted when safe.

Across the cohort, no clinically significant procedure-related complications were recorded—specifically, no bleeding requiring intervention, pneumothorax requiring tube drainage, hypoxemia requiring escalation, hemodynamic instability, or need for advanced airway/support. Chart review revealed no post-procedural infections documented in the available records. When minor mucosal oozing occurred during sampling, it was controlled intraprocedurally (e.g., suction/cold saline) and did not require escalation; per the predefined safety endpoints, such self-limited events were not classified as complications.

### 3.4. Biomarkers

As for biomarkers, p63 was positive in 45 patients (67.2%) and negative in 22 (32.8%). PD-L1 was positive in 35 cases (52.2%) and negative in 32 (47.8%). Regarding EGFR mutation testing, 54 patients (80.6%) had no mutation, while 1 patient (1.5%) had an exon 18 mutation, 7 patients (10.4%) had an exon 19 mutation, and 5 patients (7.5%) had an exon 21 mutation. All biomarker determinations (PD-L1, p63, TTF-1, synaptophysin [SYN], and Ki-67) were performed by immunohistochemistry on formalin-fixed, paraffin-embedded (FFPE) tissue. TTF-1 supports pulmonary origin and is frequently expressed in pulmonary adenocarcinoma and neuroendocrine carcinomas. Synaptophysin (SYN) marks neuroendocrine differentiation and aids in classifying neuroendocrine tumors. The Ki-67 proliferation index reflects tumor growth fraction and tends to be higher in aggressive histologies.

All histopathological and molecular investigations were ultimately performed on tissue obtained endoscopically; EBUS-TBNA was used in 42/67 patients (62.7%) for confirmation and mediastinal staging, whereas the remaining patients were diagnosed through endobronchial forceps biopsies obtained during bronchoscopy.

### 3.5. Presenting Symptoms

Presenting symptoms reflected both respiratory and constitutional complaints. Cough and weight loss were universal (100% each). Chest pain and fever were also common (83.6% each), whereas dyspnea and hemoptysis were present in 62.7% of patients. Loss of appetite and sweating were likewise reported by 62.7% each, while wheezing was less frequent (31.3%). This symptom profile aligns with the advanced-stage predominance (stage III–IV) observed in our cohort. The full distribution is summarized in Table 3 (N = 67).

### 3.6. Associations with TNM Stage

Analyzing the distribution of patients by smoking status and TNM stage among non-smokers, there were 3 cases (4.5%) in stage I, 1 case (1.5%) in stage II, 7 cases (10.4%) in stage III, and 13 cases (19.4%) in stage IV. Among smokers, 2 cases (3.0%) were in stage I, 5 cases (7.5%) in stage II, 7 cases (10.4%) in stage III, and 29 cases (43.3%) in stage IV. The Chi-square test did not indicate a statistically significant association between smoking status and TNM stage (*p* = 0.274) as seen in Table 4.

Regarding occupational exposure, patients without exposure were distributed as follows: 3 cases (4.5%) in stage I, 4 cases (6.0%) in stage II, 5 cases (7.5%) in stage III, and 15 cases (22.4%) in stage IV. Patients with occupational exposure included 2 cases (3.0%) in stage I, 2 cases (3.0%) in stage II, 9 cases (13.4%) in stage III, and 27 cases (40.3%) in stage IV. Fisher’s exact test showed no significant association between occupational exposure and TNM stage (*p* = 0.391).

The PD-L1 expression analysis revealed the following distribution: PD-L1-negative patients included 5 cases (7.5%) in stage I, 4 cases (6.0%) in stage II, 6 cases (9.0%) in stage III, and 17 cases (25.4%) in stage IV. PD-L1-positive patients had 0 cases (0.0%) in stage I, 2 cases (3.0%) in stage II, 8 cases (11.9%) in stage III, and 25 cases (37.3%) in stage IV. The Chi-square test showed a statistically significant difference (*p* = 0.026).

For the p63 marker, the distribution was as follows: p63-negative patients included 3 cases (4.5%) in stage I, 2 cases (3.0%) in stage II, 4 cases (6.0%) in stage III, and 13 cases (19.4%) in stage IV, while p63-positive patients had 2 cases (3.0%) in stage I, 4 cases (6.0%) in stage II, 10 cases (14.9%) in stage III, and 29 cases (43.3%) in stage IV. Fisher’s exact test showed a significant association between p63 status and TNM stage (*p* = 0.002).

The analysis of the relationship between chest pain and TNM stage showed that patients without chest pain were distributed in early stages, with 5 cases (7.5%) in stage I and 6 cases (9.0%) in stage II, and no cases in stages III or IV. In contrast, patients with chest pain were exclusively found in advanced stages, with 14 cases (20.9%) in stage III and 42 cases (62.7%) in stage IV. The Chi-square test demonstrated a statistically significant association between the presence of chest pain and TNM stage (*p* < 0.0001).

## 4. Discussion

This study evaluated the feasibility and safety of a bronchoscopy-based diagnostic and staging algorithm, supported by EBUS-TBNA, for patients with suspected lung cancer in a regional oncology setting. Diagnostic tissue was obtained for all patients without the need for surgical sampling, and no clinically significant complications were recorded. These findings highlight the potential of a minimally invasive approach to streamline lung cancer work-up and accelerate treatment decisions.

TNM staging provides the anatomic framework for prognostication and treatment selection in lung cancer [8]. In our cohort, the predominance of stage III–IV underscores the clinical value of an endoscopic, minimally invasive pathway—bronchoscopy with selective EBUS-TBNA—to obtain adequate tissue and establish stage early in the diagnostic work-up.

Across the cohort, no clinically significant procedure-related complications were recorded. In particular, we did not observe clinically relevant bleeding, pneumothorax requiring tube drainage, hypoxemia requiring escalation, or hemodynamic instability. These findings support the safety of a bronchoscopy- and EBUS-guided pathway in routine care and align with published data reporting high diagnostic yield and low complication rates for modern flexible bronchoscopy and EBUS-TBNA. Airway and oxygenation strategies may influence safety during EBUS-TBNA. A recent randomized controlled trial reported that performing advanced bronchoscopy with EBUS through a laryngeal mask airway (LMA) or using high-flow nasal cannula (HFNC) reduced desaturation and complications compared with low-flow nasal cannula [14].

Previous studies have demonstrated that bronchoscopy combined with EBUS-TBNA can provide accurate diagnosis and mediastinal staging in lung cancer patients, with high safety profiles. International guidelines increasingly recommend minimally invasive endoscopic techniques over surgical exploration for mediastinal evaluation. However, the implementation of these recommendations remains inconsistent in some regional centers, including parts of Eastern Europe. Our results support the growing evidence that a bronchoscopy-centered algorithm can achieve adequate tissue sampling for histopathological, immunohistochemical, and molecular analyses, aligning with best practice standards.

Several recent studies support our findings. Divisi et al. conducted a prospective multicenter study including 217 NSCLC patients, reporting 90% accuracy in mediastinal staging with EBUS-TBNA and no major complications [12]. Khoury et al. demonstrated adequacy rates of 90.1% for PD-L1 testing in a recent cohort of lung cancer patients, indicating reliable molecular profiling capabilities [13]. Additionally, recent technical advancements in EBUS, including elastography and miniforceps, have been shown to enhance diagnostic yield and safety, as discussed by Biondini et al. [15]. Our cohort’s outcomes align well with these data, reinforcing the role of EBUS-TBNA in safe, effective diagnostic and molecular evaluation in regional oncology practice.

Our findings also confirm the high procedural adequacy of bronchoscopic and EBUS-guided sampling for histopathological and molecular characterization. In our cohort, all patients underwent successful PD-L1, p63, and EGFR testing without the need for surgical biopsy, supporting the diagnostic completeness of this minimally invasive algorithm. This is particularly relevant in light of recent studies such as Khoury et al., who demonstrated that EBUS-TBNA yields adequate PD-L1 samples in over 90% of cases, enabling reliable molecular profiling and therapeutic decisions [13]. Such performance, replicated in our regional center, highlights the readiness of peripheral institutions to adopt personalized oncology protocols based solely on endoscopic sampling.

Our findings are consistent with recent evidence. A multicenter prospective study demonstrated high diagnostic yield and safety when using EBUS-TBNA in patients with prior oncologic treatment, confirming its robustness and minimal invasiveness even in complex cases [15]. Moreover, a randomized trial comparing 19G versus 22G needles reported comparable tissue adequacy for PD-L1 and other molecular markers without raising complications [16,17,18]. These data support our results, showing that our protocol achieved reliable histopathological and molecular diagnoses efficiently and safely.

This study has several limitations. First, the retrospective, single-center design and relatively small sample size may limit the generalizability of the findings. Second, the absence of a control group undergoing surgical confirmation of mediastinal staging precludes a direct comparison of diagnostic accuracy. Third, we did not assess long-term outcomes or survival impact following the application of this algorithm. Despite these limitations, the study provides valuable pilot data supporting the feasibility of a bronchoscopy-centered approach in a regional oncology setting. Such strategies may ultimately improve patient outcomes and resource utilization in lung cancer care.

In this study, “safety” referred to immediate and early procedure-related outcomes (clinically significant bleeding, pneumothorax requiring tube drainage, hypoxemia requiring escalation, hemodynamic instability, and tolerance to local anesthesia/mild sedation). Long-term survival and post-procedural pain, fever, and inflammatory or oxidative-stress markers were not systematically captured due to the retrospective design and the primary focus on diagnostic feasibility. Complete laboratory data (CBC, CRP, total protein, albumin, globulin) were not uniformly available across the cohort and are therefore not presented; these endpoints will be prospectively incorporated in future work.

## 5. Conclusions

This study demonstrated that a bronchoscopy-centered diagnostic and staging algorithm, supported by EBUS-TBNA, is feasible, safe, and effective in a Romanian regional oncology setting. The approach enabled accurate histopathological and molecular diagnosis without the need for surgical confirmation, potentially reducing diagnostic delays and optimizing resources. These results support the integration of minimally invasive strategies in similar regional centers, while further prospective studies are warranted to confirm these findings.

## Figures and Tables

**Table 1:** Baseline clinical and demographic characteristics.

Parameter	Value	Patients	
		n	%
Gender	Male	40	59.7
	Female	27	40.3
Residence	Urban	41	61.2
	Rural	26	38.8
Smoking status	Active/former	43	64.2
	Never	24	35.8
Occupational exposure	Yes	40	59.7
	No	27	40.3
Comorbidities *	None	14	
	COPD	23	
	Heart disease	13	
	Diabetes mellitus	10	
	asthma	7	

* Percentages for individual comorbidities are not shown because multiple conditions may coexist (sum > 100%).

**Table 2:** Distribution of TNM stages, histological subtypes, and diagnostic groups.

Parameter	Value	Patients	
		n	%
TNM stages	I	5	7.5
	II	6	9.0
	III	14	20.9
	IV	42	62.7
Histological subtypes	Adenocarcinoma	25	37.3
	Squamous cell carcinoma	15	22.4
	Small-cel carcinoma	14	20.9
	Neuroendocrine tumors	13	19.4
Diagnostic groups	G1a	27	40.3
	G1b	15	22.4
	G2	14	20.9
	G3	11	16.4

**Table 3:** Presenting symptoms (N = 67).

Parameter	Value	Patients	
		n	% of Total
Symptom	Cough	67	100.0
	Dyspnea	42	62.7
	Hemoptysis	42	62.7
	Chest pain	56	83.6
	Wheezing	21	31.3
	Weight loss	67	100.0
	Loss of appetite	42	62.7
	Sweating	42	62.7
	Fever	56	83.6

**Table 4:** Distribution of patients according to smoking status, occupational exposure, and tumor biomarkers by TNM stage.

Parameter	Value	TNM Stage				*p*
		I	II	III	IV	
Smoked status	NO	3 (4.5%)	1 (1.5%)	7 (10.4%)	13 (19.4%)	0.274 *
	YES	2 (3.0%)	5 (7.5%)	7 (10.4%)	29 (43.3%)	
Professional exposure	NO	3 (4.5%)	4 (6.0%)	5 (7.5%)	15 (22.4%)	0.391 **
	YES	2 (3.0%)	2 (3.0%)	9 (13.4%)	27 (40.3%)	
PD-L1	NO	5 (7.5%)	4 (6.0%)	6 (9.0%)	17 (25.4%)	0.026 *
	YES	0 (0.0%)	2 (3.0%)	8 (11.9%)	25 (37.3%)	
p63	NO	3 (4.5%)	2 (3.0%)	4 (6.0%)	13 (19.4%)	0.002 **
	YES	2 (3.0%)	4 (6.0%)	10 (14.9%)	29 (43.3%)	
Chest pain	NO	5 (7.5%)	6 (9.0%)	0 (0.0%)	0 (0.0%)	<0.0001 *
	YES	0 (0.0%)	0 (0.0%)	14 (20.9%)	42 (62.7%)	

* Chi-square test, ** Fisher’s Exact Test. The values with bold are statistically significant.

## Data Availability

The authors declare that the data of this research are available from the corresponding authors upon reasonable request.

## Abbreviations

The following abbreviations are used in this manuscript:

EBUS-TBNA	Endobronchial Ultrasound-Guided Transbronchial Needle Aspiration
NSCLC	Non-Small Cell Lung Cancer
SCLC	Small Cell Lung Cancer
PD-L1	Programmed Death Ligand 1
EGFR	Epidermal Growth Factor Receptor
ALK	Anaplastic Lymphoma Kinase
TNM	Tumor, Nodes, Metastases Staging System
CT	Computed Tomography
COPD	Chronic Obstructive Pulmonary Disease
